# Degradation of the Organochlorinated Herbicide Diuron by Rainforest Basidiomycetes

**DOI:** 10.1155/2020/5324391

**Published:** 2020-10-06

**Authors:** Caroline Henn, Ricardo M. Arakaki, Diego Alves Monteiro, Mauricio Boscolo, Roberto da Silva, Eleni Gomes

**Affiliations:** ^1^ITAIPU Binacional, Divisão de Reservatório-MARR.CD, PR, Brazil, Avenida Tancredo Neves, 6731, CEP 85856-970 Foz do Iguaçu, Paraná, Brazil; ^2^Laboratório de Bioquímica e Microbiologia Aplicada, Instituto de Biociências, Letras e Ciências Exatas, Universidade Estadual Paulista, SP, Brazil, Rua Cristóvão Colombo, 2265, Jardim Nazareth, São José do Rio Preto, São Paulo CEP 15054-000, Brazil; ^3^Departamento de Química e Ciências Ambientais, Instituto de Biociências, Letras e Ciências Exatas, Universidade Estadual Paulista, SP, Brazil, Rua Cristóvão Colombo, 2265, Jardim Nazareth, São José do Rio Preto, São Paulo CEP 15054-000, Brazil

## Abstract

The main organochlorinated compounds used on agricultural crops are often recalcitrant, affecting nontarget organisms and contaminating rivers or groundwater. Diuron (N-(3,4-dichlorophenyl)-N′,N′-dimethylurea) is a chlorinated herbicide widely used in sugarcane plantations. Here, we evaluated the ability of 13 basidiomycete strains of growing in a contaminated culture medium and degrading the xenobiotic. Dissipation rates in culture medium with initial 25 mg/L of diuron ranged from 7.3 to 96.8%, being *Pluteus cubensis* SXS 320 the most efficient strain, leaving no detectable residues after diuron metabolism. *Pycnoporus sanguineus* MCA 16 removed 56% of diuron after 40 days of cultivation, producing three metabolites more polar than parental herbicide, two of them identified as being DCPU and DCPMU. Despite of the strong inductive effect of diuron upon laccase synthesis and secretion, the application of crude enzymatic extracts of *P. sanguineus* did not catalyzed the breakdown of the herbicide in vitro, indicating that diuron biodegradation was not related to this oxidative enzyme.

## 1. Introduction

Worldwide ecosystems have received million tons of synthetic compounds, especially pesticides, since the development of chemical synthesis processes with agricultural and industrial purposes. Often heavily toxic and recalcitrant, they contaminate the cultivable soils, as well as surface and groundwater. Despite the persistence and adverse effects in human and environmental health, several pesticides are still increasingly used [1, 2].

Phenylurea herbicides are among the most frequently used agrochemicals for weed control in sugarcane, citrus, and coffee plantations. Diuron (N-(3,4-dichlorophenyl)-N,N-dimethylurea; CAS 330-54-1) is one of those pesticides having high permeability in soils, low susceptibility to natural attenuation, and strong toxicity. In addition, toxic metabolites with genotoxic and teratogenic properties, such as 3,4-dichloroanilines (3,4-DCA), N-3,4-dichlorophenylurea (DCPU), and N-(3,4-dichlorophenyl)-N-methylurea (DCPMU) can result from biotic and abiotic chemical transformations [3, 4].

Several authors have reported that phenylurea herbicide dissipation in the environment is predominantly a biological process. Considering the complexity and the high financial costs for restoration of polluted sites by means of physical and chemical treatments, bioremediation can be a promising approach to decontaminate impacted sites [5]. Some basidiomycetes can be powerful tools for this purpose, because they produce enzymes, like laccases (Lac, EC 1.10.3.2), manganese-dependent peroxidases (MnP, EC 1.11.1.13), and lignin peroxidases (LiP, EC 1.11.1.14); these enzymes have versatile catalytic mechanism, breaking down carbon linkages with aromatic ring fission, removing aryl-alkyl groups and lateral chains [6]. Thus, they can oxidize a wide range of phenolic and non-phenolic substrates, such as polycyclic aromatic hydrocarbons (PAHs) [7], dyes [8], pesticides [9], chlorophenols [10], and chlorolignin from residues of paper pulping [11].

Complete mineralization of diuron can be achieved by bacterial strains. Some strains grow by using the herbicide as the only carbon source [12, 13]. Previous studies have reported the potential of white-rot fungi to perform the biotransformation of phenylurea herbicides. However, the degradation ability is limited in this group. Little is known about complete diuron consumption by fungi, and biotransformation activity leaves several metabolites. The metabolism is assumed to be slow and mediated by cytochrome-P450, with chlorinated aromatic rings remaining intact in pure cultures [14, 15]. Here, we analyzed some aspects of the metabolism of tropical rainforest fungal strains with high capacity for diuron degradation, including possible relationships with ligninolytic enzyme production.

## 2. Materials and Methods

### 2.1. Fungal Strains

We used 13 basidiomycete strains isolated from decaying wood in Atlantic semideciduous forest fragments, at “Noroeste Paulista Ecological Station”, São José do Rio Preto/Mirassol, Northwestern São Paulo, Brazil [16]. Fungal cultures were preserved in sterile distilled water [17].

### 2.2. Inoculum

Cell fragments were scraped from fungal cultures in agar plates, under sterile conditions, and transferred to 250 mL Erlenmeyer flasks loosely caped with cotton plugs, containing 50 mL of the synthetic medium [18]. The carbon source was glucose (10 g/L), and 25 mM of N were supplied as NH_4_NO_3_. After 6-7 days growing at 28 ± 2°C, the medium was removed, and the mycelium washed twice with Knapp buffer [15]. Afterwards, fungal biomass was grinded with the buffer in a sterile mixer, and cell suspension was diluted to reach the optical density of 0.5 at the wavelength of 550 nm. This standardized suspension was used as inocula in further experiments.

### 2.3. Culture Conditions

For diuron degradation experiments, commercial formulation of Diuron Karmex® (DuPont), 80% (w/w) was used in culture medium. The herbicide was diluted in methanol (12.5 mg.mL^−1^) and homogenized by two sonication pulses of 15 minutes, following centrifugation at 10,000 g for 10 min at 10°C. Then, the solution was sterilized by filtration through a 0.22 *μ*m Millipore membrane. Aliquots were dispensed into sterile 125 mL Erlenmeyer flasks in a laminar flow. After solvent evaporation, each flask received 25 mL of sterile liquid culture medium [18], supplemented with 10 g/L of glucose, resulting in final concentration of 25 mg/L of diuron. Nitrogen was supplied in culture medium as NH_4_NO_3_ in order to create conditions of nitrogen sufficiency (25 mM of N) or nitrogen deficiency (2.5 mM of N). Diuron degradation and ligninolytic enzyme production were evaluated during 40 days of static cultivation in the dark at 28 ± 2°C. At the end of the cultivation period, cultures were centrifuged at 10,000 g for 10 min. In order to assess the adsorption to biomass, part of the harvested mycelia was triturated in a mortar after centrifugation, extracted twice with 1 volume of n-hexane, and the combined extracts dried at 45°C. The residue was resuspended with 1 mL of the HPLC mobile phase, filtered in a 0.22 *μ*m membrane and analyzed. The remaining mycelia were dried at 70°C for biomass determination.

### 2.4. HPLC and LC-MS Analysis

Samples of filtered culture medium or mycelia extracts were cleaned up using C_18_, 500 mg Agilent Sampli-Q cartridges. 10 mL of centrifuged culture medium were passed throughout cartridges using a manifold and a vacuum pump. Elution employed 5 mL of methanol, followed by 2.5 mL of acetonitrile. The degradation rates of diuron were estimated using a Flexar Perkin-Elmer HPLC (binary pump and UV-Vis detector adjusted to 240 nm), with the program Chromera 3.4. Separations were performed injecting 10 *μ*L of cleaned samples in a C_18_ Agilent Zorbax column (4.6 × 250 mm), kept in an oven at 40°C and elution was done in isocratic mode (1 mL.min^−1^), with a mobile phase of acetonitrile and water (60/40 v/v), with 0.1% of formic acid. Then, diuron with 99% purity (UltraChem), DCPU, and DCPMU (Sigma) were used as standards to build the calibration curve in HPLC. All samples were injected three times, and results are presented as means.

To identify metabolites, a Perkin-Elmer Flexar SQ 300MS mono quadrupole with a ESI source was used. 20 *μ*L of cleaned samples were injected, and LC separations were done as described above. The mass selective detection, from 40 to 300 m/z, was done in the positive ionization mode. Operational parameters were drying gas flow of 12 L/min, at 350°C. Nebulizer gas pressure was 60 psi; capillary voltage was 115 V and 30 V for the skimmer. Mass spectra in the full scan mode were obtained by scanning from 40 to 300 m/z. The associated software was SQ300 MS Detector Driver V1.1.

### 2.5. Enzyme Assays

Enzymatic activities in the crude extract of fungal cultures were determined spectrophotometrically by reactions carried out at 40°C. Laccase was measured based on ABTS (Sigma) oxidation (*ε* = 3.6.10^4^ M^−1^ cm^−1^), by reading the oxidized product at 420 nm [19]. The lignin peroxidase activity was determined using veratryl alcohol (Fluka) oxidation to veratraldehyde (*ε* = 9.103 M^−1^ cm^−1^) at 310 nm [20]. Manganese-dependent peroxidase had activity determined by generating lactate-Mn^3+^ complexes (*ε* = 8.1.10^3^ M^−1^ cm^−1^), detected at 240 nm [21, 22]. Thus, 1 unit of the enzymatic activity was expressed as the amount of enzyme capable of oxidizing 1 *μ*mol of substrate per minute under assay conditions and expressed as U/g of dry biomass.

### 2.6. Assay for Enzyme-Mediated Degradation of Diuron

The potential of fungal laccase to degrade diuron was tested using a 10 mL reaction system, containing diuron in a final concentration of 20 mg/L, in 50 mM sodium acetate buffer at pH 2.5 and 0.03% ABTS. The volume of the crude enzyme used was 10% (v/v). For this assay, extracellular culture fluids were previously treated for 15 min with a solution of bovine catalase (24.5 U.mL^−1^) to remove hydrogen peroxide. Assays lasted for 24 hours using the best physical-chemical parameters of pH (2.5) and temperature (55°C) previously determined for this laccase. As controls, reaction mixtures replacing the enzyme extract by water were incubated under identical conditions. Periodically, aliquots were taken, disposed in 1.5 mL capped tubes, and frozen immediately in liquid nitrogen. For HPLC analysis, samples were melted and filtered through 0.22 *μ*m membranes.

### 2.7. Other Analytical Procedures

Reducing sugars were determined by the DNS method [23], using *D*-glucose as the analytical standard.

### 2.8. Data Analysis

We used one-way ANOVA followed by Tukey posthoc tests to test the effect of fungal strains on diuron removal from the culture medium. Normality and homoscedasticity were tested using Shapiro-Wilk and Levene's test, respectively. Analyses were conducted in the software R Studio version 3.5.1 [24].

## 3. Results and Discussion

### 3.1. Diuron Degradation by Basidiomycete Strains

The use of fungal strains for bioremediation usually rely on the concept of cometabolism: the existence of an auxiliary source of carbon and nitrogen as a condition for microbial development, allowing secondary metabolism enzymes to degrade xenobiotic compounds. The physiological purposes of these enzymes are diverse from catabolism of these molecules, useless as carbon and energy sources [25–27]. In fact, the 13 basidiomycete strains evaluated here could not act upon diuron otherwise, since any of them could grow with the herbicide as the sole carbon and energy source (0 to 75 mg/L, 20 days—data not shown).

Once glucose was the carbon and energy source, the capacity of degrading diuron varied significantly among strains. Only 6 out of the 13 strains dissipated diuron in 20-day-old cultures (Table 1). Diuron degradation ranged from 7.3% to 96.8%, being *Pluteus cubensis* SXS 320 the most active, followed by *Hexagonia hirta* MCA 131 (19.3%) and *Pycnoporus sanguineus* MCA16 (19.3%). Sorption of herbicide to mycelium was negligible, as observed by chromatographic analysis of the mycelia extract (data not shown). These degradation rates are uncommon for isolated basidiomycete strains, and few works have reported similar performance [28–30].

The mycelial development of the fungi was, in general, negatively affected by the herbicide. However, for *Polyporus* sp. MCA 128 and *Datronia stereoides* MCA 167, the mycelial biomass increased 5 and 6 times in the diuron-containing medium, when compared to the control, but these strains did not degrade the herbicide (Table 1). Literature reports divergent fungal behavior on this aspect. Similar results were observed for *Mycelia sterilia* INBI 2-26 and interpreted as a response against toxicological stress [31], while the growth of *Mortierella* strain was not affected by diuron [29].

**Figure 1:**
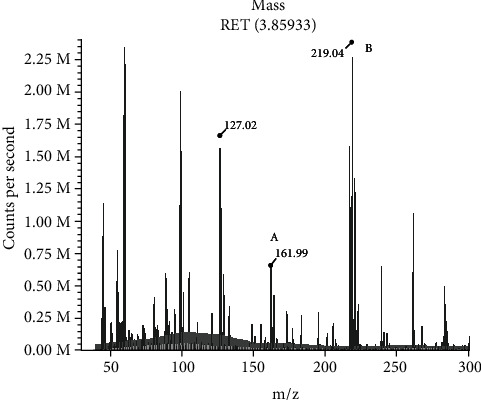
LC-MS fragment profiles of metabolites from the fungal activity. (a) 3,4-DCA. (b) DCPMU.

Residual metabolites were found in the crude extract of five out of the six strains that degraded diuron, identified in HPLC analysis as DCPMU and DCPU, being the first one, derived from the demethylation of diuron, accumulated in a greater extent. LC-MS confirmed the identity of DCPMU (m/z 219.04) and revealed the presence of 3,4-DCA (m/z 161.99) as an additional metabolite (Figure 1). This pattern appears to be similar to *Sphingomonas* sp. SRS2 and the fungus *Mortierella* sp. LEJ701, whose metabolism raised DCMPU as the first product, followed by DCPU and 3,4-DCA, as later metabolites [29, 32]. This model is in agreement with the degradation route involving successive demethylations of the parental molecule, proposed by Sørensen [29].

Previous works [3] found a high toxicity of these mono and di-demethylated metabolites when compared to the parental diuron molecule. Bonnet et al. [33], measuring bioluminescence of *Vibrio fischeri* after 15 minutes of incubation, demonstrated that DCPMU and DCPU are 3.7 times more toxic than diuron, whereas 3,4-DCA was 109 times more disrupting of homeostasis. Therefore, it is clear that both the time from the arising of each compound and the accumulation of aromatic final residues, like 3,4-DCA, are key aspects for the election of bioremediation agents.

**Figure 2:**
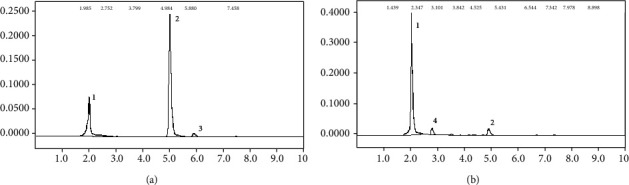
Chromatograms from 20-day-old cultures of *P. cubensis* SXS 320. (a) abiotic control. (b) culture with initial 25 mg/L of diuron. Peak 1: unknown compound from the culture medium. Peak 2: diuron. Peak 3: residue of commercial formulation. Peak 4: compound from normal fungal metabolism (present in the controls without herbicide).

On the other side, no traces of DCPU or DCPMU, as well no 3,4-DCA, were found in *P. cubensis* SXS 320 culture extracts. Precipitation as insoluble compounds is improbable, since the solubility of all known diuron metabolites in water is higher than the parental molecule [34]. Therefore, the absence of new peaks suggest that the aromatic ring was effectively broken down (Figure 2(b)). These results mean that this basidiomycete strain is unique, since diuron metabolism by this group is usually incomplete, producing the demethylated residues described above [30, 35]. The peak 3, an unknown aromatic residue present in commercial formulation of pesticide, disappeared after the *P. cubensis* SXS 320 growth, similarly to diuron (Figure 2).

Different mechanisms can be involved in intra- or extracellular fungal metabolism of aromatic compounds. Oxidation often is the first step, leading to the generation of quinones and arene oxides [14]. Quinones are the product of lignin peroxidases, manganese-dependent peroxidases, or laccases, referred as the ligninolytic system, while arene oxides result from the cytochrome P450 monooxigenase activity [7]. The oxidation increases the polarity, solubility, and reactivity, favoring subsequent metabolic routes towards conjugation and elimination [14]. The fungal strains evaluated here, although unable to mineralize the entire molecule (except for *P. cubensis* SXS 320), can metabolize it to mono and di-demethylated compounds, which may become unavailable by bounding to humic acids or can be mineralized by other indigenous microorganisms [15, 36], such as *Rodococcus* sp. and *Pseudomonas* sp. These microorganisms can grow by using diuron as their sole carbon source, in metabolic routes carried out by enzymes codified in plasmids [12, 13].

*P. cubensis* SXS 320 could degrade diuron with no evidences of accumulation of aromatic residues. However, some steps must pass through the 3,4-DCA biotransformation, perhaps involving cell bound enzymes like cytochrome P450 monooxygenases, similar to those found in *Phanerochaete chysosporium* [2, 25, 36]. As chromatographic analysis were performed only for 20-day-old cultures, it could be late to found the possible intermediaries, such as 3,4-dichloroacetanilide or N-(3,4-dichlorophenyl)-*β*-ketoglutanyl-*δ*-amide. This strain has a very promising profile and deserves future investigation for bioremediation purposes.

### 3.2. Diuron Biotransformation×Laccase

The synthesis of ligninolytic enzymes has been associated to the biodegradation of aromatic pollutants. Because many aromatic xenobiotics share common structural features with lignin substructures, both can be attacked by unspecific oxidative free-radicals resulting from the lignin-degrading enzymatic activity, in a cometabolic pattern that depends on additional carbon and energy sources [25, 26]. In this study, we found a significant influence of diuron upon the enzyme activity, in a clear process on induction (Table 1). Laccase synthesis was strongly induced by the herbicide, reaching 240 U/g of dry biomass for *Pycnoporus sanguineus* MCA16, which was 25 times higher when compared to the diuron-free medium. For *P. tenuiculus* MCA11, activity reached 82 U/g of dry biomass (induction of 20 times compared to control), and for unidentified Agaricales MCA17, laccase reached 231 U/g, a level 58 times higher than control. Despite the strong effect upon laccase synthesis and secretion, diuron metabolism lacks connection to extracellular laccase, manganese peroxidases, or lignin peroxidases yields in the culture medium for the basidiomycetes studied. The two most efficient degrader strains, *H. hirta* MCA 131 and *P. cubensis* SXS 320, did not produce any detectable ligninolytic enzyme. Fungal strains that carried out some diuron dissipation while producing some ligninolytic enzyme synthesized only laccases (*P. sanguineus* MCA16, *Datronia caperata* MCA 5, and *P. tenuiculus* MCA11). Neither manganese peroxidase nor lignin peroxidase were produced in measurable quantity by any fungal isolate (Table 1).

**Figure 3:**
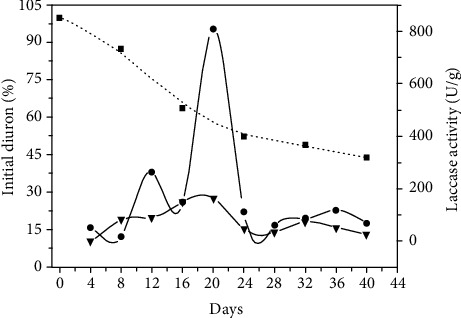
Profile of laccase production and diuron degradation by *P. sanguineus* MCA 16. (■) residual diuron level in cultures; (•) laccase activity in medium with initial 25 mg/L of diuron; (▼) laccase activity in control medium (without herbicide).

The induction of the synthesis and secretion of ligninolytic enzymes by xenobiotics has been extensively described [25, 26]. However, the linkage between enzymatic levels and the effective biotransformation of xenobiotic compounds is less clear. Some studies reported increased metabolism of xenobiotics associated with higher ligninolytic enzyme levels as a result of induction. An example of this is atrazine degradation by *Coriolus hirsutus*, *Coriolopsis fulvocinerea,* and *Cerrena maxima* [37], or the biodegradation of diuron by the laccase of *Ganoderma lucidum* [38]. Conversely, no evidences of these enzymes acting upon xenobiotics were found in *Flammulina velupites* and *Phanerochaete velutina,* also degrading diuron, or *Pleurotus ostreatus* and *Bjerkandera adusta* degrading PAHs [9, 39]. The synthesis of ligninolytic enzymes by the strains evaluated here lacks direct connection with rates of diuron degradation, both in quantitative and temporal scales (Table 1 and Figure 3). Because laccase yields in the culture medium were not a premise for herbicide degradation, further investigations were done in vitro, searching for the possible roles of the extracellular enzyme in the process.

**Figure 4:**
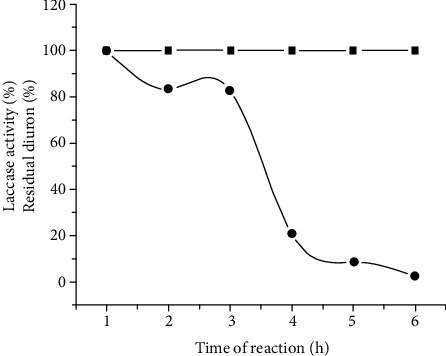
Diuron persistence during in vitro degradation reaction, carried out with crude extracts with laccase from *P. sanguineus* MCA 16 and ABTS as a mediator (pH 2.5, 55°C). (■) residual diuron; (•) remaining laccase activity.

Crude enzyme extracts consisting of filtered 20-day-old cultures of *P*. *sanguineus* MCA 16 were used for an in vitro assay in the presence of ABTS as mediator (Figure 4). Laccase retained 80% of the initial activity after 3 hours of incubation. However, no changes on initial diuron concentration were observed after 24 hours, neither new products were detected on HPLC analysis of reaction mixtures, despite the tolerance of the enzyme to the previously determined best catalytic conditions (pH 2.5 and 55°C).

Laccases are versatile enzymes that degrade a wide range of aromatic compounds. In vitro, HBT, ABTS, and TEMPO are common synthetic mediators used to study the degradation of aromatics like PAHs and PCBs [10]. Conversely, aryl alcohols and humic acids can act as laccase mediators in natural conditions [10, 37]. Once we employed ABTS, the most common laccase mediator, with no results, it suggests that diuron degradation by *P. sanguineus* MCA16 is not directly related to laccases or other enzymes secreted to the medium, at least at the first steps of herbicide biodegradation.

**Figure 5:**
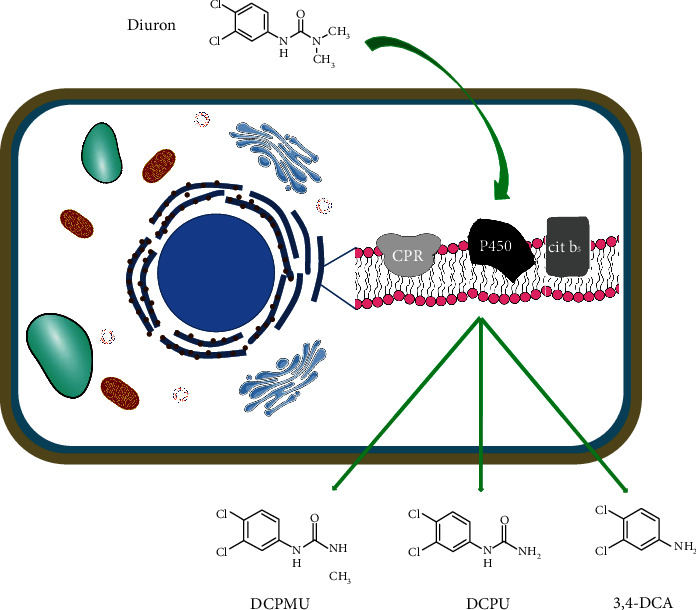
Possible intracellular route of diuron biodegradation in fungal cells, involving cytochrome P450, found in the endoplasmic reticle membrane.

The well described diuron biodegradation pathways involve successive N-demethylations, giving rise to DCPMU and DCPU, followed by amide bond hydrolysis, which results in 3,4-DCA [36]. Bacterial biodegradation of diuron is usually carried out by hydrolases, codified by plasmids [36]. Fungal strains, on the other side, depend on extracellular or intracellular oxidative enzymes to deal with the herbicide; this is the case of laccases and cytochrome P450 (CYP). The last ones, heme-thiolate proteins, act mainly as monooxygenases, but can also undergo hydroxylation, dealkylation, deamination, and dehalogenation of the substrates [40]. Once extracellular laccases were not implicated in the biodegradation of diuron, citochrome P450 enzymes probably are the catalytic agents involved (Figure 5).

Cytochrome P450 enzymes are widespread and highly diversified among fungal species, in order to meet the metabolic needs and environmental challenges. These enzymes are classified into more than 400 families and recognized by the ability of acting on primary and secondary metabolism, as well as on the degradation of xenobiotics and general detoxification processes [41]. The involvement of CYP isoforms in the phenylurea metabolism is well documented for plants, like artichoke (*Helianthus tuberosus*) and ginseng (*Panax ginseng*) [42, 43]. Recently, the role of CYP enzymes in the biodegradation of phenylureas by basidiomycete species was demonstrated; when the cytochrome P450 inhibitors like PB (piperonil botoxide) and ABT (1-aminobenzotriazole) were added to the culture medium, the biodegradation of diuron by *Ganoderma lucidum* dropped from 35 to 31 and 22%, respectively [38]. Strong inhibition effects were also observed for *Phanerochaete chrysosporium* [2] and *Trametes versicolor* [44] when ABT was added to the culture medium containing diuron.

### 3.3. Modulation of Fungal Metabolism by Nitrogen

Nitrogen availability in the culture medium is often the main factor involved in lignin (and aromatics) metabolism by basidiomycetes. Early findings demonstrated that low nitrogen levels were related to high ligninolytic enzymes titers, with a consequent gain on the efficiency of xenobiotics biotransformation. Together, evidences based mainly on *Phanerochaete chrysosporium* metabolism suggested that nitrogen starvation was a premise for ligninolytic enzyme production [45]. This proved to be true in experiments dealing with pollutants like polycyclic aromatic hydrocarbons (PAHs) [46] and dyes [47]. However, there is still no consensus on such pattern. Often, the best conditions for aromatic biotransformation depend only on nitrogen levels in the culture medium, or the presence of ligninases, with no relation between these two variables [29, 48].

**Figure 6:**
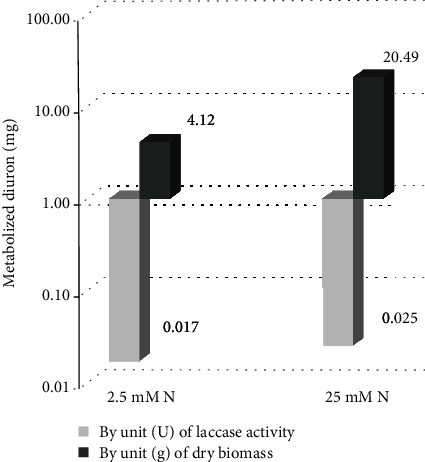
Diuron degradation by *P. sanguineus* MCA 16, according to biomass and laccase in low- and high-nitrogen culture conditions.

Diuron degradation by *P. sanguineus* MCA16 in a medium with 25 mM of N reached 39% in 20 days. This is 3 times higher than those growing under nitrogen starvation. The difference was 5-fold when considering the amount of diuron degraded per biomass unit (Figure 6). Conversely, the herbicide degraded per unit of the laccase activity was quite similar (0.017 and 0.025 mg.U^−1^). Although all evidences point out to cell-bounded mechanisms to explain diuron metabolism by *P. sanguineus* MCA 16—perhaps involving cytochrome P450 oxidation—it is not possible to discard laccase acting a secondary degradation step. This is because organic pollutants sometimes undergo initial oxidations, reductions, and conjugations inside cells, being exported as intermediaries susceptible to extracellular laccases and peroxidases [25].

**Figure 7:**
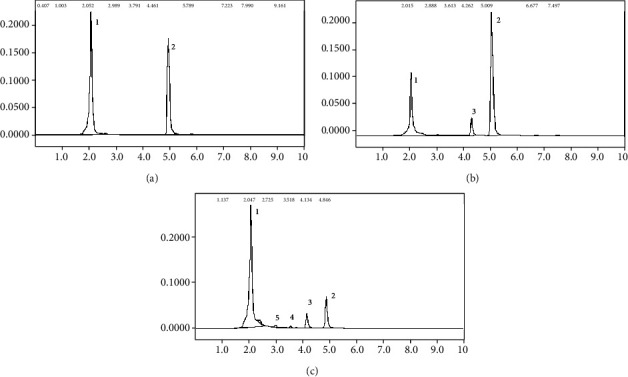
Chromatograms from culture medium of *P. sanguineus* MCA 16, with initial 25 mg/L of diuron. (a) abiotic control. (b) 20-day culture, in the presence of 2.5 mM of N. (c) 40-day culture, with 25 mM of N. Peak 1: unknown compound from culture medium. Peak 2: diuron. Peaks 3 and 5: DCPMU and DCPU, respectively. Peak 4: unknown metabolite.

The different levels of nitrogen provided in the culture medium leaded to modifications in the qualitative profile of diuron metabolism by *P. sanguineus* MCA16, besides the quantitative effects. In the case of nitrogen rich cultures, chromatographic profiles displayed 2 additional peaks (peaks 4 and 5, Figure 7(c)), when compared to the cultures with 2.5 mM of nitrogen, having only DCPMU (Figure 7(b)). The peak 5 is DCPU, but the peak 4, a compound with a retention time between that of DCPU and DCPMU and generated only under nitrogen sufficiency, could not be identified. Changes in the metabolic pathways of fungi dealing with aromatics, as response to nitrogen availability, are registered for several strains. As discussed by Aydin et al. [49], the metabolism of phenanthrene by *Phanerochaete chrysosporium* was found to change according to nitrogen availability; under nitrogen-rich conditions, the main metabolites were *trans*-dihydrodiols; nitrogen starvation, on the other side, led to the emergence of quinones and other oxidation products. When testing the ability of *Aspergillus niger* and *Phlebiopsis* cf. *ravanelli* of decolorize synthetic dyes, Sweety et al. [50] found that while increasing concentrations of carbon sources (glucose or lactose) in the culture medium favored the biodegradation, nitrogen (supplied as ammonium sulphate or ammonium nitrate), when abundant (>0.5%), inhibited the decolorization of crystal violet and orange G-II. The role on nitrogen availability determining the metabolic pathways needs further exploration, what has been achieved recently through genomic and proteomic approaches [51].

## 4. Conclusions

The basidiomycete strains studied showed high potential to be used in bioremediation, mainly *P. cubensis* SXS 320, that degraded 96% of diuron, leaving no aromatic residues. Laccase seems not to be involved in the degradation of diuron by cometabolism as supposed, despite the strong induction of the enzyme in the presence of this herbicide. Nitrogen availability in the culture medium proved to be a key factor for the degradation of diuron.

## Figures and Tables

**Table 1:** Growth, laccase production, and diuron degradation by basidiomycetes after 20 days of cultivation in medium containing initial 25 mg/L of diuron, 2.5 mM of N, and 10 g/L of glucose. (–) = nondetectable. Superscript numbers (1, 2, and 3) refer to statistically significant groups.

Strain	Control		Diuron 25 mg/L		
	Lac (U/g)	Biomass (g/L)	Lac (U/g)	Biomass (g/L)	Degradation (%)
*Gloeophyllum striatum* MCA 2	—	0.93 ± 0.02	—	0.67 ± 0.00	—
*Datronia caperata* MCA 5	34.1 ± 6.68	0.80 ± 0.08	45.24 ± 1.50	0.50 ± 0.08	12.1 ± 6.2^2.3^
*Trametes modesta* MCA 6	—	1.76 ± 0.23	—	0.18 ± 0.03	—
*Gloeophyllum striatum* MCA 7	—	1.3 ± 0.00	—	0.98 ± 0.01	—
*Polyporus tenuiculus* MCA 9	—	0.97 ± 0.01	—	0.37 ± 0.03	7.3 ± 3.7^2.3^
*Polyporus tenuiculus* MCA 11	4.14 ± 0.0	1.72 ± 0.66	82.13 ± 17.9	1.06 ± 0.29	16.3 ± 2.0^2^
*Pycnoporus sanguineus* MCA 16	9.51 ± 9.48	1.47 ± 0.12	240.6 ± 76.2	0.79 ± 0.03	19.3 ± 3.1^2.3^
Agaricales n. i. (MCA 17)	3.94 ± 1.25	0.8 ± 0.02	231.0 ± 5.49	0.2 ± 0.00	—
*Polyporus sp.* MCA 128	—	5.07 ± 0.12	—	24.7 ± 22.6	—
*Hexagonia hirta* MCA 131	—	2.11 ± 0.30	—	1.08 ± 0.44	19.3 ± 1.4^2^
*Datronia stereoides* MCA 167	—	5.52 ± 0.04	—	39.4 ± 25.3	—
*Pluteus cubensis* SXS 320	—	1.40 ± 0.23	—	1.52 ± 0.28	96.8 ± 0.8^1^
SXS 323 *Dacryopinax elegans*	—	2.39 ± 0.00	—	1.64 ± 0.35	—

## Data Availability

The data used to support the findings of this study are included within the article. Additional details can be provided by the corresponding author on request.
